# Respiratory tract virus infections in the elderly with pneumonia

**DOI:** 10.1186/s12877-019-1125-z

**Published:** 2019-04-16

**Authors:** Matti Aronen, Laura Viikari, Ia Kohonen, Tytti Vuorinen, Mira Hämeenaho, Maarit Wuorela, Mohammadreza Sadeghi, Maria Söderlund-Venermo, Matti Viitanen, Tuomas Jartti

**Affiliations:** 1 Department of Geriatrics, Turku City Hospital, Turku, Finland; 2 Department of Radiology, Turku University Hospital, Turku, Finland; 3 Department of Medical Microbiology, Turku University Hospital and Institute of Biomedicine, University of Turku, Turku, Finland; 4 Department of Virology, University of Helsinki, Helsinki, Finland; 5 Department of Pediatrics and Adolescent Medicine, University of Turku and Turku University Hospital, PO Box 52, 20520 Turku, Finland; 6 Pori, Finland

**Keywords:** Elderly, Etiology, Influenza virus, Parainfluenza virus, Pulmonary disease, Respiratory, Respiratory syncytial virus, Rhinovirus, Virus

## Abstract

**Background:**

In children suffering from severe lower airway illnesses, respiratory virus detection has given good prognostic information, but such reports in the elderly are scarce. Therefore, our aim was to study whether the detection of nasopharyngeal viral pathogens and conventional inflammatory markers in the frail elderly correlate to the presence, signs and symptoms or prognosis of radiographically-verified pneumonia.

**Methods:**

Consecutive episodes of hospital care of patients 65 years and older with respiratory symptoms (*N* = 382) were prospectively studied as a cohort. Standard clinical questionnaire was filled by the study physician. Laboratory analyses included PCR diagnostics of nasopharyngeal swab samples for 14 respiratory viruses, C-reactive protein (CRP) and white blood cell count (WBC). Chest radiographs were systematically analysed by a study radiologist. The length of hospital stay, hospital revisit and death at ward were used as clinical endpoints.

**Results:**

Median age of the patients was 83 years (range 76–90). Pneumonia was diagnosed in 112/382 (29%) of the studied episodes. One or more respiratory viruses were detected in 141/382 (37%) episodes and in 34/112 (30%) episodes also diagnosed with pneumonia. Pneumonia was associated with a WBC over 15 × 10^9^/L (*P =* .006) and a CRP value over 80 mg/l (*P <* .05). A virus was detected in 30% of pneumonia episodes and in 40% of non-pneumonia episodes, but this difference was not significant (*P* = 0.09). The presence of a respiratory virus was associated with fewer revisits to the hospital (*P* < .05), whereas a CRP value over 100 mg/l was associated with death during hospital stay (*P* < .05). Respiratory virus detections did not correlate to WBC or CRP values, signs and symptoms or prognosis of radiographically-verified pneumonia episodes.

**Conclusion:**

Among the elderly with respiratory symptoms, respiratory virus detection was not associated with an increased risk of pneumonia or with a more severe clinical course of the illness. CRP and WBC remain important indicators of pneumonia, and according to our findings, pneumonia should be treated as a bacterial disease regardless of the virus findings. Our data does not support routine virus diagnostics for the elderly patients with pneumonia outside the epidemic seasons.

**Electronic supplementary material:**

The online version of this article (10.1186/s12877-019-1125-z) contains supplementary material, which is available to authorized users.

## Background

The number of over 80-year-old patients with weaning immune system is rising rapidly in Western societies, but the clinical significance of respiratory virus infections among this group remains unclear. The burden of pneumonia among the elderly is high as it includes significant morbidity, mortality and costs around the world [1]. In the United States alone, about 85% of the deaths caused by pneumonia or influenza occur in the age group of 65 years and older, and in 2013, in the health care of patients of all ages, more than 16.1 billion dollars were spent on pneumonia [2].

As polymerase chain reaction (PCR) and rapid antigen detection tests are increasingly available for respiratory virus detection [3], currently in 13 to 30% of lower respiratory tract infections among the elderly, a virus has been implicated [4, 5]. In the age group of 65 years and older, influenza virus (Flu), respiratory syncytial virus (RSV) and parainfluenza virus (PIV) are the leading viral causes of respiratory morbidity and mortality [6–8], while other viral causes include rhinovirus (RV) and coronaviruses (CoV) [9]. However, in contrast to pediatric data, there are only a few reports concerning the usefulness of respiratory virus diagnostics in the elderly, and virus diagnostics have shown only limited value in reducing antibiotic use and the length of hospital stay [10, 11]. While influenza detection is seen as an important diagnostic tool by the physicians, the detection of other viruses is seen less useful [11].

Among the elderly, the all-cause mortality rate associated with respiratory viral infection increases with age and is approximately 6–7% among the over 85-year-old subjects [8]. This may, however, be an underestimate due to a general bias towards predominantly recording influenza as a death course, while neglecting other virus infections. Other common risk factors of a severe respiratory viral infection among the elderly include underlying medical conditions and poor response to influenza vaccine [12, 13]. In adults, mixed infections with a respiratory virus and a bacterial pathogen, especially rhinovirus with pneumococci, have been shown to associate with more severe pneumonia and longer hospitalization period [14–17]. C-reactive protein (CRP) value and white blood cell count (WBC) seem to be insufficient methods in differentiating sole bacterial, mixed and sole virus pneumonias, although no unambiguous methods exist [18, 19].

All of the aforementioned findings support a clinically meaningful role of virus diagnosis among the frail elderly. Thus, the aim of this study was to investigate how viral pathogens detected in the nasopharynx and conventional inflammatory markers (WBC and CRP) correlate to signs, symptoms and prognosis of pneumonia among the age group of 65 years and over. We hypothesized that virus detection could give clinically relevant information in addition to conventional inflammatory markers and chest radiograph findings in treating frail elderly patients.

## Methods

### Study design

This prospective follow-up study investigated the association between virus detection and predefined clinical outcomes in elderly hospitalized patients. STROBE criteria were respected.

### Subjects

The study was carried out in geriatric wards of the Turku City Hospital between July 2007 and April 2009 as part of a previously introduced study [20]. Consecutive Turku residing patients of over 65 years of age suffering from respiratory symptoms requiring hospital admission were recruited in this study. Patients were excluded from the study if they were in extremely poor condition, had severe dementia or had been quarantined. The patient or his/her trustee was informed about the study both orally and in a written form. Patient’s previously named trustee was approached if patient’s ability to independent decision making was deteriorated. A written consent from the patient or his/her trustee was required to participate in the study. The study protocol was approved by the Ethics Committee of the Turku University Hospital and it complies with the ethical rules for human experimentation stated in the Declaration of Helsinki.

### Clinical follow-up

Patients were considered to have respiratory symptoms if they had coryza, cough, sore throat, hoarseness or nasal stuffiness. In pneumonia episodes, the need for oxygen was considered as a sign of dyspnea. At study entry patients or their trustees were interviewed using a standardized questionnaire (Additional file 1: “Questionnaire”), which included questions concerning the form of living before hospitalization, the hospital unit the patient was coming from, chronic diseases, influenza vaccination status, height, weight, smoking habits and physical activity. Hospital records were reviewed for the clinical history and gender of the patient. Having one or more of the following conditions was defined as having other diseases: dementia, depression, diabetes, rheumatic disease or history of cancer.

The length of the hospital stay, hospital revisit and death during the hospital stay were used as clinical outcomes of this study. Patient was discharged from the ward when the illness no longer required hospital treatment. A new episode of hospital care between 2 weeks and six months from the last visit was considered a revisit; earlier visits were considered prolonged illness and later a separate episode. For this study only hospital revisits in which respiratory symptoms were present were recorded.

### Diagnostics

Treatment-related chest radiographs taken in the study hospital were systematically analysed in a blinded fashion by a study radiologist. The presence of interstitial infiltrate and/or lobar atelectasis in the chest radiograph was considered pneumonia after congestive heart failure as an etiology was excluded.

From all patients meeting the inclusion criteria, nasopharyngeal swab samples were collected (sterile flocked swab, 520CS01, Copan, Brescia, Italy) by study physicians within 24 h of admission. The swabs were then stored in dry tubes in a refrigerator for a maximum of 24 h before transportation to the laboratory where they were stored at − 80 °C. The swab samples were analysed at the Department of Virology, University of Turku, Turku, Finland by a multiplex reverse-transcriptase (RT-)PCR test (Seeplex RV12 ACE Detection; Seegene, Seoul, Korea) for adenovirus, coronavirus NL63 and OC43, human bocavirus, human metapneumovirus (MPV), influenza A and B, and PIV1–3, and by using an ‘in-house’ RT-PCR test for RSV, RV - including rhinovirus type C - and enteroviruses (EVs) [21]. Based on our previous experiences in amplicon sequencing, if the in-house PCR test could not distinguish enteroviruses from rhinoviruses, the result was considered rhinovirus positive [22, 23]. HBoV infections were serologically confirmed to be acute infections at the Department of Virology, University of Helsinki, Helsinki, Finland.

Blood samples for CRP and WBC analysis were routinely collected from all the patients as part of hospital treatment. The serum samples were stored at − 80 °C and analyzed by the hospital laboratory. The highest values measured during the hospital stay were used in statistical analysis.

### Statistics

In basic statistics, two sample *t*-test, χ^2^ test and Fischer exact test (when counts < 5) were used when appropriate. Logistic regression with full model was used to analyse the association between clinical outcomes and virus etiology, pneumonia, chronic illnesses (cardiovascular diseases, respiratory diseases, other diseases), age, gender and laboratory findings (WBC, CRP). Statistical significance was established at the level of *P <* .05. For statistics SAS Enterprise Guide 4.3 (SAS Institute Inc., Cary, NC, USA) was used.

## Results

### Study population

**Fig. 1:**
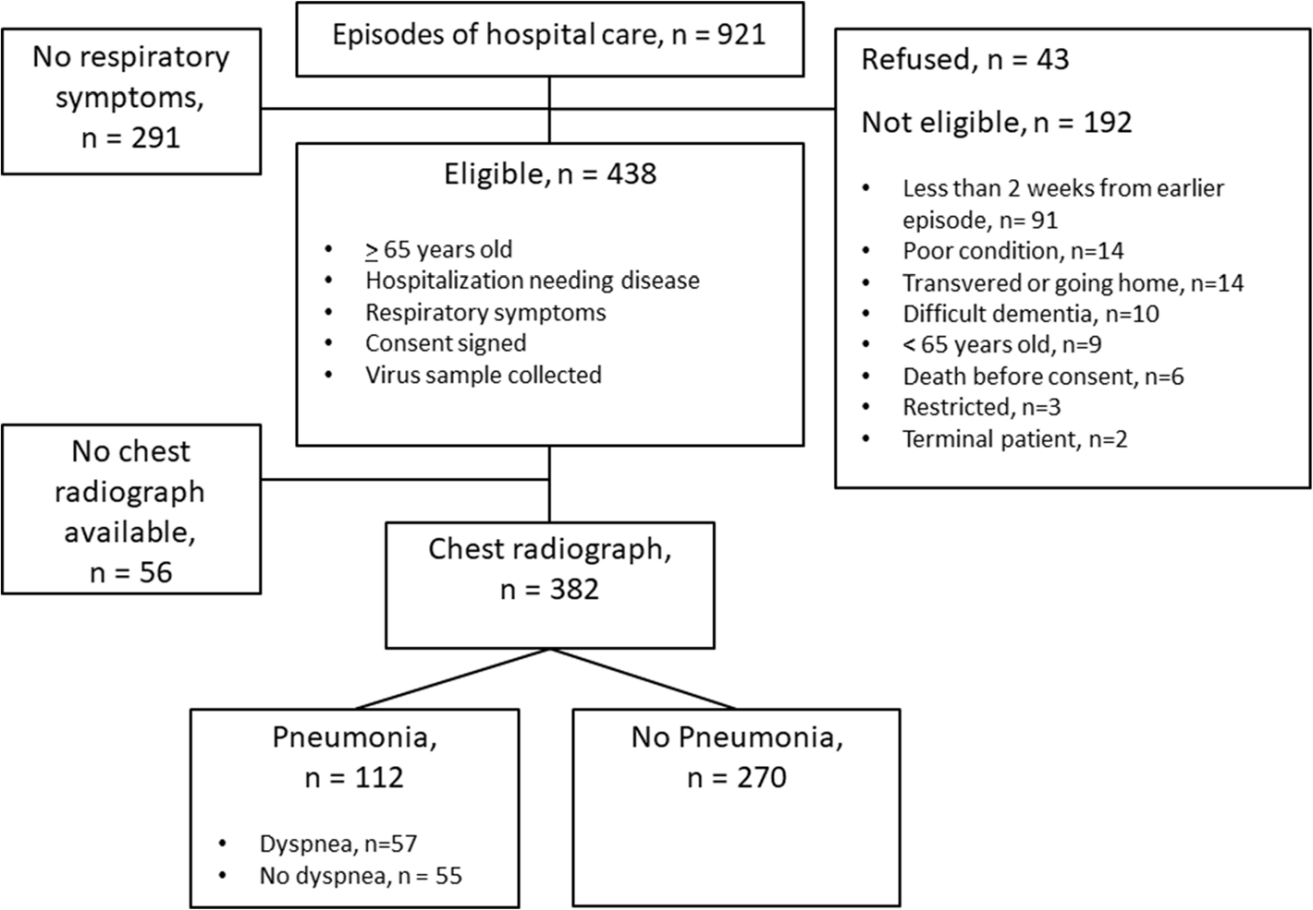
Study flow chart

A total of 921 episodes of hospital care were screened (Fig. 1). Of those 921 screened episodes of hospital care 438 fulfilled the initial study requirements of age 65 years or over, hospitalization-needing disease, respiratory symptoms and a signed consent to participate in the study. A swab and serum samples were collected from the patients of these 438 episodes. Of the 438 episodes, a chest radiograph was available for 382 episodes. In 112 of the 382 episodes the patient was diagnosed from a chest radiograph finding as having pneumonia. In 55 (49%) of these 112 pneumonia episodes the patient had pneumonia with dyspnea and in 57 (51%) pneumonia without dyspnea. The characteristics (age, gender, presence of chronic illnesses, smoking status) of 56 patients who had respiratory symptoms but no chest radiograph available, were not different from the included subjects (*P* > .08, data not shown).

### Patient characteristics

Mean age of the patients in the study was 82.9 (sd 7.2) years (Table 1). A diagnosis of cardiovascular disease was present in 74% and respiratory disease in 34% of the study episodes. There were more men (57%) in the group diagnosed with pneumonia than in the group not diagnosed with pneumonia (42%) (*P =* .008). The weight of the patients seemed to be lower in the group diagnosed with pneumonia (*P =* 0.05), whereas heart dysrhythmia seemed to be more common among patients not diagnosed with pneumonia (*P =* .03). In connection with the study episodes without pneumonia, in 16% the patient had a history of a stroke or transient ischemic attack (TIA) and in 16% the patient smoked, compared to 9.2 and 25% of the study episodes diagnosed with pneumonia, respectively. These differences were, however, not statistically significant (*P* = .08 and *P =* .1, in the same order). Otherwise cardiovascular, respiratory and other diseases were equally common in the study episodes diagnosed with pneumonia and the study episodes not diagnosed with pneumonia (all *P >* .1).

**Table 1:** Patient Characteristics

	Respiratory Symptoms (*n* = 382)		
	Pneumonia (*n* = 112)	No Pneumonia (*n* = 270)	*P*-value
Gender (male/female)	64/48 (57%/43%)	114/156 (42%/58%)	**0.008**
Age	83 (7)	83 (7)	0.8
Weight	65 (15)	69 (17)	***0.05***
Respiratory diseases	41/109 (38%)	90/262 (34%)	0.5
- Asthma/COPD	32/109 (30%)	76/262 (29%)	0.9
- Other lung disease	11/109 (10%)	29/262 (11%)	0.9
Cardiovascular diseases	80/109 (73%)	202/262 (77%)	0.4
- Stroke/TIA	10/109 (9.2%)	42/262 (16%)	0.08
- Heart dysrythmia	23/109 (21%)	84/262 (32%)	***0.03***
- Myocardial infarction	18/109 (17%)	38/262 (15%)	0.6
- Heart failure	24/109 (22%)	69/262 (26%)	0.4
- Hypertension	52/109 (48%)	120/262 (46%)	0.7
- MCC	34/108 (31%)	87/261 (33%)	0.7
Other diseases	65/105 (62%)	183/256 (71%)	0.07
- Dementia	22/105 (21%)	55/256 (21%)	0.9
- Depression	5/105 (4.8%)	20/256 (7.8%)	0.3
- Rheumatic disease	25/105 (24%)	72/256 (28%)	0.4
- Diapetes mellitus			0.4
- Type 1	0/105 (0%)	2/256 (0.8%)	
- Type 2	19/105 (23%)	58/256 (18%)	
- Cancer status			0.2
- Terminal	2/105 (1.9%)	9/256 (3.5%)	
- Have been treated	7/105 (6.7%)	32/256 (13%)	
- Under treatment	6/105 (5.7%)	20/256 (7.8%)	
Smoking	20/81 (25%)	31/192 (16%)	0.1

Data expressed as n (%) or mean (standard deviation)

Two sample *t*-test, χ^2^ test and Fischer exact test (when counts < 5) were used

Significant values are shown bold and italic

*COPD*, chronic obstructive pulmonary disease, *TIA* Transient ischemic attack, *MCC* Morbus coronarius cordis

### Virus detection

A respiratory virus was detected in 141/382 (37%) nasopharyngeal swabs of the study episodes (Table 2). Overall, rhinovirus and influenza virus were the two most common viruses detected, both present in 35 (9%) study episodes, followed by parainfluenza in 28 (7%), coronavirus in 24 (6%), RSV in 22 (6%), MPV in 8 (2%) and adenovirus in 2 (1%) cases. During one study episode, prolonged bocavirus shedding was found in the nasopharynx of the patient, but serology did not confirm an acute infection. In 30% of the study episodes diagnosed with pneumonia a respiratory virus was also detected, whereas the same was true for 40% of the study episodes not diagnosed with pneumonia. This association was, however, not statistically significant (*P* = 0.09). Virus detection was also found not to be associated with dyspnea (*P* > .5), nor were there virus-specific differences found between study episodes diagnosed with pneumonia and with dyspnea, and study episodes diagnosed with pneumonia but without dyspnea (*P* > .1).

**Table 2:** Presence of respiratory viruses in episodes with pneumonia and dyspnea

	Respiratory symptoms (*n* = 382)		*P*-value	Pneumonia (*n* = 112)		*P*-value
	Pneumonia (*n* = 112)	No pneumonia (*n* = 270)		Dyspnea (*n* = 57)	No dyspnea (*n* = 55)	
Rhinovirus	12 (11%)	23 (8.5%)	0.5	8 (15%)	4 (7.0%)	0.2
Influenza virus	7 (6.3%)	28 (10.4%)	0.2	2 (3.6%)	5 (8.8%)	0.4
Parainfluenza virus	5 (4.5%)	23 (8.5%)	0.2	2 (3.6%)	3 (5.3%)	1.0
Coronavirus	7 (6.3%)	17 (6.3%)	1.0	4 (7.3%)	3 (5.3%)	0.7
Respiratory syncytial virus	3 (2.7%)	19 (7.0%)	0.1	3 (5.5%)	0 (0%)	0.1
Human metapneumovirus	3 (2.7%)	5 (1.9%)	0.7	2 (3.6%)	1 (1.8%)	0.6
Adenovirus	1 (0.89%)	1 (0.37%)	0.5	1 (1.8%)	0 (0%)	0.5
Bocavirus	1 (0.89%)	0 (0%)	*Na*	1 (1.8%)	0 (0%)	0.5
1 or more viruses	34 (30%)	107 (40%)	0.09	18 (33%)	16 (28%)	0.6
2 or more viruses	7 (6.3%)	13 (4.8%)	0.6	4 (7.3%)	3 (5.3%)	0.7

χ < 2 test and Fischer exact test (when counts < 5) were used

Data expressed as n (%)

### White blood cell count and CRP-reactive protein level

The mean WBC value was 13.8 × 10^9^/L in study episodes with pneumonia and 11.1 × 10^9^/L in study episodes without pneumonia, but this difference was not statistically significant (*P* = 0.07, Table 3). As a categorical variable, WBC over 15 × 10^9^/L was associated with study episodes diagnosed with pneumonia (*P <* .006, Table 3).

**Table 3:** White blood cell count and C-reactive protein values in episodes with pneumonia and dyspnea

	Respiratorys symptoms (382)		*P*-value	Pneumonia (112)		*P*-value
	Pneumonia (112)	No pneumonia (270)		Dyspnea (57)	No dyspnea (55)	
WBC	13.8 (sd 14)	11.1 (sd 9.3)	0.07	12.4 (sd 5.7)	15.1 (sd 19)	0.3
WBC over 10	57 (51%)	111 (42%)	0.08	27 (47%)	30 (56%)	0.4
WBC over 15	***29 (26%)***	***38 (14%)***	***0.006***	14 (26%)	15 (26%)	1.0
CRP	***146 (sd 92)***	***105 (sd 79)***	***< 0.0001***	158 (sd 95)	134 (sd 88)	0.2
CRP over 80	***81 (72%)***	***151 (56%)***	***0.003***	43 (78%)	38 (67%)	0.2
CRP over 100	***71 (63%)***	***120 (44%)***	***0.0007***	38 (69%)	33 (58%)	0.2

Data expressed as mean (standard deviation) or n (%)

Two sample t-test, χ2 test were used

Significant values are shown bold and italic

*WBC* White blood cell count (×10^9^/L), *CRP* C-reactive protein (mg/l)

The mean CRP value was 146 mg/l in study episodes with pneumonia and 105 mg/l in study episodes without pneumonia (*P* < 0.001). As a categorical variable, a CRP value over 100 mg/l, or even over 80 mg/l, was associated with a pneumonia finding in the chest radiograph (*P <* .05 for both, Table 3).

When comparing study episodes diagnosed with pneumonia and with one or more respiratory viruses, and study episodes diagnosed with pneumonia but without respiratory viruses, no differences between WBC or CRP values were found (*P >* .2, Table 4). Corresponding effects of the most common viruses (rhinovirus, influenza virus, coronavirus, RSV and parainfluenza virus) were also tested separately, and no differences were found (*P* > .1).

**Table 4:** Associations between laboratory findings, clinical outcomes, pneumonia and presence of a virus in hospital episodes with respiratory symptoms

	Pneumonia+/virus+ (1)	Pneumonia+/virus- (2)	Pneumonia−/virus+ (3)	Pneumonia−/virus- (4)	P (1vs2)	P (1vs3)	P (3vs4)	P (1vs4)
WBC over 15	7 (21%)	22 (29%)	8 (7.5%)	30 (19%)	0.4	***0.03***	***0.01***	0.8
CRP over 100	24 (71%)	47 (60%)	40 (37%)	80 (49%)	0.3	***0.001***	0.06	***0.02***
Had a revisit	11 (32%)	26 (33%)	32 (30%)	67 (41%)	0.9	0.8	0.06	0.3
Over 13 nights at ward	11 (32%)	23 (29%)	28 (26%)	59 (36%)	0.8	0.5	0.08	0.7
Death at ward	1 (2.9%)	10 (13%)	7 (6.5%)	11 (6.8%)	0.1	0.4	0.9	0.4

χ^2^ test and Fischer exact test (when counts < 5)

Significant values are shown bold and italic

*Pneumonia +* Episodes with pneumonia, *Pneumonia-* Episodes without pneumonia, *Virus +* Episodes with a virus detected, *Virus-* Episodes without a virus detected, *CRP* C-reactive protein (mg/l), *WBC* White blood cell count (×10^9^/L)

### Clinical outcomes

A clinical outcome was available for 357 of the 382 study episodes. Of the 357 study episodes with clinical outcome available, in 108, the patient stayed in the hospital for more than 13 nights, in 127, the patient had a revisit and in 29, the patient died during the hospital stay (Table 5).

**Table 5:** Patient characteristics of those who died at ward

Case number	Age range	Gender	Pneumonia	Dyspnea	Respiratory disease	Cardiovascular disease	Other disease	A respiratory virus	Over 13 nights at ward	WBC over 15	CRP over 100	Human metapneumovirus	Adenovirus	Rhinovirus	Influenza virus	Respiratory syncytial virus	Parainfluenza virus	Coronavirus
1	70–80	M	0	1	0	1	1	1	1	0	1	0	0	0	0	0	0	1
2	90–100	M	0	1	1	1	1	1	1	0	1	0	0	0	0	0	1	0
3	80–90	F	0	1	1	0	1	1	1	1	0	0	0	0	0	0	1	0
4	90–100	F	0	1	0	1	1	1	1	0	0	0	0	0	0	0	1	0
5	90–100	F	0	1	0	1	1	1	0	0	0	0	0	0	0	0	1	0
6	90–100	F	1	0	0	1	*Na*	1	1	0	1	0	0	1	0	0	0	0
7	90–100	M	0	1	0	1	1	1	1	0	1	0	0	1	0	0	0	0
8	80–90	M	0	0	0	0	1	1	1	0	0	1	0	0	0	0	0	0
9	80–90	F	1	0	0	0	*Na*	0	1	1	1	0	0	0	0	0	0	0
10	70–80	M	1	1	0	0	0	0	1	1	1	0	0	0	0	0	0	0
11	70–80	M	0	1	0	1	*Na*	0	1	1	1	0	0	0	0	0	0	0
12	80–90	M	0	0	*Na*	*Na*	*Na*	0	1	1	1	0	0	0	0	0	0	0
13	80–90	F	0	1	1	1	1	0	1	1	1	0	0	0	0	0	0	0
14	80–90	M	0	1	0	0	0	0	0	1	1	0	0	0	0	0	0	0
15	90–100	F	0	1	0	1	0	0	0	1	1	0	0	0	0	0	0	0
16	70–80	M	1	1	1	1	0	0	1	0	1	0	0	0	0	0	0	0
17	90–100	M	1	1	0	1	0	0	1	0	1	0	0	0	0	0	0	0
18	80–90	F	0	0	*Na*	*Na*	*Na*	0	1	0	1	0	0	0	0	0	0	0
19	70–80	M	0	1	1	0	1	0	1	0	1	0	0	0	0	0	0	0
20	80–90	F	1	1	0	1	1	0	0	0	1	0	0	0	0	0	0	0
21	80–90	F	1	0	0	1	1	0	0	0	1	0	0	0	0	0	0	0
22	70–80	M	1	1	0	0	0	0	0	0	1	0	0	0	0	0	0	0
23	70–80	M	0	1	1	0	1	0	0	0	1	0	0	0	0	0	0	0
24	80–90	M	0	1	0	0	1	0	0	0	1	0	0	0	0	0	0	0
25	80–90	M	0	1	1	1	1	0	0	0	1	0	0	0	0	0	0	0
26	80–90	M	1	1	1	1	1	0	1	0	0	0	0	0	0	0	0	0
27	80–90	F	0	0	0	1	1	0	1	0	0	0	0	0	0	0	0	0
28	80–90	F	1	1	*Na*	*Na*	*Na*	0	0	0	0	0	0	0	0	0	0	0
29	90–100	M	1	0	1	1	1	0	1	1	0	0	0	0	0	0	0	0
	Σ		11		9	17	17	8	19	9	21	1	0	2	0	0	4	1
	%		42		35	65	74	31	73	31	81	4	0	8	0	0	15	4

Significant values are shown bold and italic

*Σ* Sum of a column, *%* Column sum percentage, *WBC* White blood cell count (×10^9^/L), *CRP* C-reactive protein (mg/l), *M* Male, *F* Female, *1* Present, *0* Not present, *Na* Data not available

In study episodes diagnosed with pneumonia, the presence of a respiratory virus was neither associated with clinical outcomes (i.e. over 13-night hospital stay, number of revisits or death at ward) nor with WBC values over 15 × 10^9^/L or CRP values over 100 mg/l (all *P* > .1, Table 4). Similar results were also seen with lower WBC and CRP cutoff values of 10 × 10^9^/L and 80 mg/l, respectively (data not shown). Also in connection with study episodes diagnosed with pneumonia, there was no association between the above mentioned clinical outcomes and laboratory findings (WBC over 15 × 10^9^/L/l or CRP over 100 mg/l) (*P* > .2). In study episodes diagnosed with pneumonia and with dyspnea, death at ward was seen in 15% of the study episodes, whereas in connection to study episodes diagnosed with pneumonia but without dyspnea, the same was true in 5.3% of the study episodes, although this difference was not statistically significant (*P* = 0.07). However, study episodes diagnosed with pneumonia and with dyspnea lasted longer than study episodes diagnosed with pneumonia but without dyspnea (*P* = .02).

No difference in the number of deaths at ward was seen between study episodes diagnosed with pneumonia and study episodes not diagnosed with pneumonia. A negative association was found between hospital revisit and virus detection; a revisit was less probable when a virus was present than when a virus was not present; 43 (31%) revisits occurred among the virus-positive study episodes and 93 (39%) revisits among the virus-negative study episodes (*P* < .05, Table 6). Finally, a CRP value over 100 mg/l was associated with death at ward; 21 of the 29 (72%) deceased patients had CRP values over 100 mg/l (*P* = .04. Table 6).

**Table 6:** Association between CRP and leukocyte values and main viral findings and clinical outcomes

Variable	Over 13 nights at ward			Had a revisit			Exitus at ward		
	OR	95% limits		OR	95% limits		OR	95% limits	
Pneumonia	0.767	0.449	1.309	0.818	0.491	1.360	0.886	0.336	2.337
Leuk >15	1.303	0.690	2.460	1.336	0.726	2.461	1.158	0.376	3.565
CRP > 100	1.279	0.785	2.084	1.104	0.691	1.764	**2.845**	**1.021**	**7.933**
Virus	0.736	0.450	1.203	**0.620**	**0.385**	**0.998**	0.836	0.318	2.199
Influenza virus	0.480	0.188	1.221	0.441	0.181	1.071	–	–	–
Rhinovirus	1.508	0.685	3.320	1.153	0.525	2.530	0.421	0.052	3.378
Coronavirus	0.744	0.278	1.988	0.785	0.309	1.995	0.981	0.118	8.146

Multivariable logistic regression analysis was used

*CRP* C-reactive protein (mg/l), *WBC* White blood cell count (× 10^9^/L)

## Discussion

The study shows three main findings. Firstly, radiologically confirmed pneumonia was not associated with respiratory virus detection. Moreover, in the studied episodes of hospital care diagnosed with pneumonia, the presence of a respiratory virus was associated neither with clinical outcomes, nor with WBC or CRP values. Against our study hypothesis, all the studied episodes of hospital care in which the patient was diagnosed with one or more respiratory viruses were, in fact, associated with a less severe clinical course in terms of the number of hospital revisits. Secondly, radiologically confirmed pneumonia was associated with the indicators of a severe bacterial infection, WBC over 15 × 10^9^/L and CRP over 100 mg/l. Thirdly, a CRP value over 100 mg/l was associated with death at ward.

Our finding, namely that 30% of the study patients diagnosed with pneumonia also had a respiratory virus present in nasopharynx, is in line with recent studies executed on adults that suggest that even one third of pneumonia cases are associated with a respiratory virus [4, 5, 9]. In our study, rhinovirus was the most common virus present in the study episodes diagnosed with pneumonia, followed by coronavirus and influenza virus. Treanor et al. anticipated in their study that the role of these common cold viruses, coronavirus and rhinovirus, among the elderly will rise in the future, although PIV, RSV and influenza are still considered the most harmful viruses among the elderly [6–8]. Our findings also support this idea of a rising clinical significance of common cold viruses, especially rhinovirus, among the elderly [6, 24, 25]. An association between elevated disease severity and dual infection, especially rhinovirus/pneumococcal infection, in the adult population have been reported previously [14, 17]. Our analyses, however, showed no differences in the severity of the study episodes diagnosed with pneumonia regardless of whether there was a respiratory virus present or not.

Frailty, immunologic weakening and cardiopulmonary diseases are understood to predispose to pneumonia when a viral infection occurs [26]. In our study, a respiratory virus was found in no less than 40% of the elderly patients who suffered from respiratory symptoms but were not diagnosed with pneumonia. At the same time, the risk of a hospital revisit in all the studied episodes of hospital care seemed to be lower when a virus was present than when no virus was fund. These findings support the idea that respiratory viruses are merely innocent bystanders in patients with pneumonia [27].

Our study strengthens the idea that high CRP and WBC values are associated with pneumonia in patients with respiratory symptoms but have limited value as independent predictors [28]. In adult populations, only relatively high CRP values have been shown useful in predicting the presence of pneumonia, and a cut-off value of 100 mg/l is mentioned in some studies [29]. Krueger et al. concluded in their CAPNETZ-study with 1337 patients aged 62 ± 18 years that WBC and CRP are higher in typical bacterial than in atypical or viral etiology community-acquired pneumonias [30]. Gao et al. showed in their study that high levels of CRP were induced as well as correlated with the complement activation in patients infected with severe influenza A [31]. In our study, we saw no difference in inflammatory markers according to virus etiology.

According to our data, among the elderly, a respiratory disease that elevates CRP to over 100 mg/l could be linked to death on the count of that in such cases pneumonia is probable. Lee et al. showed similar results in their study with 424 patients aged 70.4 +/− 15.6 years [32]. Interestingly, they also showed that in addition to CRP the albumin level was associated with a 28-day mortality in hospitalized patients with a community-acquired pneumonia. On the other hand, Ortqvist et al. saw no association between high CRP and mortality in hospital-treated pneumonia patients and Krueger et al. stated in the CAPNETZ-study that WBC in contrast to CRP increased with the severity of a community-acquired pneumonia [30, 33].

The strengths of our study include prospective design, large sample size and sensitive virus-detection methods. Also, pneumonia was radiologically confirmed. However, there are some limitations to our study as well. As the study observed hospitalization-requiring episodes among frail geriatric patients, the results cannot be generalized as such to treating outpatients. Also, the swab samples were collected from the upper airways and thus infections solely in the lower airways may have been missed. Further, as the virus samples were collected in 2007–2009, they naturally give specific information concerning those years only. The study was carried out ten years ago and in one center which limits the generalizability of the results. Many respiratory viruses exhibit a seasonal variation in temperate climates. However, at species level annual virus epidemics are relatively stable in climates with defined winter seasons like in Finland [34]. We used modern PCR diagnostics that have been in routine use ever since. We believe that nearly a 2-year recruitment period with 438 samples gives a relatively good picture of virus epidemics at species levels in our area. This study has prospective design and gives information about the effect of common respiratory virus infections, even though there is some annual variation in circulating viruses. Due to many chronic diseases in the elderly, virus-induced respiratory symptoms may be difficult to distinguish from other symptoms. However, for dyspnea, we used an objective criterion based on oxygen saturation.

This study gives valuable information about the significance of virus findings in nasopharynx and inflammatory markers among frail elderly patients with respiratory symptoms.

## Conclusions

In elderly patients, the presence of respiratory viruses in the nasopharynx seems to have limited value in assessing the severity and the short-time prognosis of the disease. If a virus is found, it may in fact indicate a clinical situation with a better prognosis. In this study, not one respiratory virus correlated with the presence, signs and symptoms or prognosis of radiographically-verified pneumonia among the elderly. However, this study did find that a CRP over 80 mg/l and a WBC over 15 × 10^9^/L were linked to pneumonia and a CRP over 100 mg/l to elevated mortality during hospital stay. All in all, this study shows that pneumonia should be treated in elderly people as a bacterial disease regardless of virus findings.

## Additional file

Additional file 1: Standard clinical questionnaire. A written form to collect information concerning the form of living before hospitalization, the hospital unit the patient was coming from, chronic diseases, influenza vaccination status, height, weight, smoking habits and physical activity. (DOCX 15 kb)

## Abbreviations

AdV: Adenovirus
CI: Confidence interval
COPD: Chronic obstructive pulmonary disease
CoV: Coronavirus
CRP: C-reactive protein
EIA: Enzyme immunoassay
EV: Enterovirus
HBoV: Human bocavirus
MMSE: Mini-Mental State Examination
MPV: Human metapneumovirus
OR: Odds ratio
PCR: Polymerase chain reaction
PIV: Parainfluenza virus
RSV: Respiratory syncytial virus
RV: Rhinovirus
TIA: Transient ischemic attack
WBC: White blood cell count
