# Supplementary material for: Respiratory tract virus infections in the elderly with pneumonia
Source: BMC Geriatr. 2019 Apr 16;19:111. doi: 10.1186/s12877-019-1125-z (PMC6469155; doi:10.1186/s12877-019-1125-z)
Supplement: Supplementary file 1 — Standard clinical questionnaire. A written form to collect information concerning the form of living before hospitalization, the hospital unit the patient was coming from, chronic diseases, influenza vaccination status, height, weight, smoking habits and physical activity. (DOCX 15 kb) [file 12877_2019_1125_MOESM1_ESM.docx]

**Potilaan nimi __________________________________________________________ Henkilötunnus ___________________________**

**Tuloarvio:**

Asumismuoto ennen sairaalaan tuloa: koti __, palveluasunto __, vanhainkoti _, pitkäaikaissairaanhoito __

Mistä tulee: tk päiv__, ksh__, TYKS ea __, sairaalasiirto __

Paino _______ kg, pituus ____ cm

Pitkäaikaissairaudet:  keuhkosairaus, mikä/mitkä ______________________________________________________________________

 sydän- ja verenkiertoelimistön sairaus, mikä/mitkä ___________________________________________________

 diabetes, mikä tyyppi ja kuinka kauan _____________________________________________________________

 sidekudostauti/reuma, mikä/mitkä ________________________________________________________________

 syöpä, mikä/mitkä _____________________________________________________________________________

 immuunipuutos, syy/mikä/mitkä _________________________________________________________________

 muu sairaus, mikä/mitkä ________________________________________________________________________

Lääkitys:________________________________________________________________________________________________________

_______________________________________________________________________________________________________________

_______________________________________________________________________________________________________________

Influenssa ja muut rokotukset:_______________________________________________________________________________________

Tupakointi:  kyllä,  ei koskaan,  lopettanut, kuinka monta vuotta sitten: ___________________________________________________

Fyysinen aktiivisuus:  vuodepot.,  avustettava,  omatoiminen, liikkuminen vähäistä,  omatoiminen, ulkoilee säännöllisesti

Ravitsemustila/nestetasapaino: MNA (oma kaavake)

Dementiaseula: MMSE (oma kaavake)

Kontakti flunssaisiin ihmisiin 2 vk ajan ennen sairaalaan tuloa:  kyllä,  ei,  ei tiedossa

Kontakti lapsiin 2 vk ajan ennen sairaalaan tuloa:  kyllä,  ei,  ei tiedossa

Syy sairaalahoitoon? ______________________________________________________________________________________________

**Patients name ________________________________________________________Social security number ___________________________**

**Entry evaluation:**

Form of living before hospitalization: home __, service home __, rest home _, institution for chronically ill patients __

Patient arrives from: health care centers emergency room__, home nursing__, Turku university hospital emergency room __, transfer between hospitals __

Weight_______ kg, Height ____ cm

Chronic illnesses:  pulmonary disease, what ______________________________________________________________________

 cardiovascular disease, what ____________________________________________________________________

 Diabetes, type and since when ___________________________________________________________________

 connective tissue / rheumatic disease, what _________________________________________________________

 cancer, type _____________________________________________________________________________

 immunodeficiency, reason/what type _________________________________________________________________

 other illness, what ________________________________________________________________________

Medication: ________________________________________________________________________________________________________

_______________________________________________________________________________________________________________

_______________________________________________________________________________________________________________

Influenza and other vaccinations: _______________________________________________________________________________________

Smoking:  yes,  never,  quit, how many years ago: _________________________________________________________________

Physical activity:  bedridden.,  assistance needing,  self-sufficient, low physical activity,  self-sufficient, daily outdoor life

Nutrition/fluid balance: MNA (separate form)

Dementia screenin: MMSE (separat form)

Interaction with individuals with flu 2 weeks pior hospitalization:  yes,  no,  not known

Interaction with children 2 weeks prior hospitalization:  yes,  no,  not known

Reason for hospitalization? ______________________________________________________________________________________________
